# The role and economics of immunotherapy in solid tumour management

**DOI:** 10.1177/1078155220963190

**Published:** 2020-10-09

**Authors:** Dominic Adam Worku, Victoria Hewitt

**Affiliations:** 1 Faculty of Medical Sciences, Oncology and Palliative Care Team, Newcastle University, Newcastle upon Tyne, UK; 2 St Georges Hospital, London, UK; 3 Graduate School, Faculty Medical Sciences, Newcastle University, UK

**Keywords:** Immunotherapy, cancer, healthcare, oncology

## Abstract

Cancer therapeutics is a rapidly changing field which offers patients the prospect of a better quality of life and cure. Immunotherapy has become a unique approach for select metastatic solid tumours. While initial results do show durable responses in select patients, there are concerns on how best to utilise this expensive resource which can result in costly side effects and in whom the use of biomarkers to stratify patients is still in its infancy. Given the ageing population and extreme challenges on healthcare, economic modelling with regards to immunotherapy is imperative especially now when it is being considered for further cancer types.

Cancer is a process whereby normal cells acquire mechanisms to circumvent the normally controlled processes of proliferation and apoptosis.^1^ Unfortunately, despite best efforts the incidence of cancer is rising, with 14 million new cases reported worldwide in 2012. Paralleling this rise is the increasing sales of oncology medications which in 2000 represented 3.5% of global pharmaceutical sales and 7% in 2011. A major contribution to this increase is the emergence of targeted therapies which can offer superior outcomes in select patients.^2–4^ Whilst radiotherapy and chemotherapy remain mainstay in the oncologist’s arsenal and act by indiscriminately killing normal and cancerous cells alike, with dose limiting toxicities. Immunotherapy works indirectly by promoting the immune system to recognise and destroy cancer cells to produce clinical benefit even in late stage and metastatic disease.^1,5^

The origins of Immunotherapy derive from the 1800 s where William Coley utilised mixtures of live and inactivated Strep.Pyogenes and Serratia to induce remission in sarcoma, lymphomas, and testicular cancer with varying success. Recently immunotherapy is being employed in select solid cancer management including malignant melanoma and non-small cell lung cancer (NSCLC) however in non-invasive bladder cancer it has been used for decades in the form of the Baccile Calmette-Guerin vaccine.^6^ While the immune system is good at detecting and eradicating tumorigenic cells through immune surveillance, established tumours employ many mechanisms to evade detection including the production of immunosuppressive cytokines and reduced surface major histocompatibility complex (MHC) expression. Immunotherapies encompass a wide range of biologically active molecules including interferons, interleukins, and immune checkpoint inhibitors (ICIs) of these the latter are the most studied, utilised, and researched.^5^ ICIs work to enhance T-cell activation which are essential for anti-tumour activity and requires two distinct signals to be present. The first of these is the interaction between the T-cell receptor (TCR) and the peptides being presented by the MHC on antigen presenting cells (APC) at the draining lymph node while the second signal is a confirmatory co-stimulation signal involving interaction between the T-cell and APC by CD28:CD80/CD86 respectively. In the absence of this costimulatory signal T-cell anergy occurs. One method by which T-cell activation is limited to avoid hyperstimulation of the immune system is by the T-cell CD28 homolog, Cytotoxic T-lymphocyte -Associated Protein 4 (CTLA-4) which is upregulated on initial T-cell activation and competitively and with greater affinity than CD28 binds to CD80/CD86 on APCs and downregulates their expression. CTLA-4 also plays an essential role in regulatory T-cells function to maintain immune tolerance.^7,8^ Therefore by inhibiting the action of CTLA-4 one potentiates T-cell activation, proliferation and infiltration of the tumour leading to potentially robust anti-tumour responses. CTLA-4 is the target of the humanized IgG monoclonal antibody, Ipilimumab, which since 2011 has been licensed for the treatment of malignant melanoma. Another key target of ICI therapy is Programmed Death Receptor (PD-1R) blockade. This is expressed on a plethora of immune cells including B, T and NK cells whose normal functions are altered in the setting of cancer. The ligand of this receptor, PD-L1 is expressed on stromal and tumour cells and upon binding downregulates T-cell activation, proliferation and IL-2 production thereby limiting anti-tumour responses. This pathway is the target of the ICIs Pembrolizumab and Nivolumab which block PD-1R.^7^

Historically Malignant melanoma had a median survival of 6 months with Dacarbazine the standard of care in patients with inoperable disease despite showing no conclusive survival benefit.^9^ In 2011 the first phase III trial to demonstrate Ipilimumab’s and immunotherapies superiority was reported. This involved 676 patients of good functional status with previously treated stage III-IV melanoma who had progressed despite best standard care. Participants were randomized to three groups each receiving four treatments: Ipilimumab with glycoprotein 100 (gp100) a peptide vaccine and adjuvant (n-403), Ipilimumab monotherapy (n-137) or gp100 alone (n-136). The median overall survival of these groups was 10.0, 10.1 and 6.4 months respectively at follow-up (p < 0.001). In addition, 45.6% and 23.5% of Ipilimumab monotherapy recipients were alive at 12 and 24 months respectively with a 36% reduction in progression noted. Importantly, on re-exposure to Ipilimumab clinical responses were still seen.^10,11^ As a result of this pivotal study Ipilimumab was approved in 2011 for advanced/unresectable melanoma as well as an adjuvant therapy for fully resected stage III disease.4 The National Institute of Clinical Excellence (NICE) appraisal however concluded that while Ipilimumab was clearly beneficial that only 30% of recipients may show some benefit and of these a minority show durable responses. As such it was deemed that without manufacturer discount with total treatment Ipilimumab would not be cost-effective. In addition, there was methodological concerns with the lack of clinical difference reported between escalating doses of Ipilimumab and the usefulness of the gp100 adjuvant.^12^ More recent work published in 2018 has highlighted the power and synergistic effect combination immunotherapy can have. In the pivotal CheckMate-067 trial looking at 3-year outcomes in those with untreated grade III/IV malignant melanoma, patients (n=945) were randomized equally to receive either monotherapy or combination therapy involving Nivolumab and Ipilimumab until either progression or unacceptable toxicity. Patients who received combination therapy had a higher objective and complete response (58% and 19% respectively) than those on monotherapy after only a median of 4 doses. This correlated with a progression free survival (PFS) of 11.5 months and an overall survival (OS) rate of 58%. This study highlighted that Nivolumab therapy was more efficacious in all patient groups than Ipilimumab with a complete response of 16% and 5% and OS of 52% and 34% respectively. Importantly these responses were durable and reinforced observations seen in earlier trials and highlighted the superior outcomes that combination therapy holds even upon extremely limited exposure. It is because of such data and the concept of cure that many patients often embrace the use of immunotherapy. However, it remains unknown if these patients are cured or if they represent immunotherapy induced tumour dormancy.^7,13^

Despite these results immunotherapies may be ineffective in up to 50% of patients, explanations include tumour heterogeneity as well resistant subclonal populations of tumour cells produced by prior therapy. Importantly there may be other immune checkpoint pathways yet unappreciated which run parallel to and circumvent those currently targeted.^14^ Unlike traditional therapies immunotherapies do not commonly cause hair loss, nausea, and infertility.^1^ Instead by leading to unopposed stimulation of the immune system, ICIs can lead to autoimmune reactions and multi-organ dysfunction through an uncontrolled, dysregulated immune response which paradoxically may lead to enhanced anti-tumour activity. Every organ system has been implicated as part of immune related adverse effects (iRAEs) with dermatological complications, hypothyroidism and hypopituitarism well described in the literature occurring up to 3 years post-exposure.^5,7,15^ Currently up to two-thirds of patients receiving anti-CTLA-4 therapy experience irAEs of which one third are gastrointestinal in nature ranging from diarrhoea to colitis and can necessitate hospital admission and therefore be resource intensive to manage. In many recipients however grade I/II toxicities are observed with 80% of side effects resolving within 4–6 weeks of corticosteroid therapy. It is these toxicities which are often underreported in clinical trials and reported economic analyses.^7,16,17^ Evidently understanding and identifying patients in whom immunotherapies will work is of paramount importance given their costs and side effect profile. At present the only biomarker used in clinical practice is tumour PD-L1 expression, which is used in NSCLC to determine the likelihood of response. However, this has several disadvantages with PD-L1 expression known to vary with time, previous treatment exposure, and underlying method of detection.^17^ In addition, there has been reports of a 20-30% response rate to anti-PD-1R therapy in patients who were PD-L1 negative making its validity questionable. It is important therefore for formal identification of novel, robust, cost-effective, and reliable biomarkers (e.g. microRNAs) to enhance immunotherapy efficacy and safety in a cost-effective manner while avoiding potential treatment delays.^12,18,19^ The cost of cancer care is currently greater than any other chronic condition and this expense cannot be explained alone by the rarity of the underlying cancer or high research and development costs or a given therapeutic agents efficacy. Indeed complicit with this are the high indirect costs including a loss of income in patients and treatment related costs due to often debilitating side-effects.^2,19,20^ A recent systematic review, looking at the cost of immunotherapy treatment complications in 844 elderly melanoma patients, receiving either immunotherapy (n = 528) or target therapy (n = 316) revealed that the overall costs in both groups suffering complications were on average four times higher than those who did not ($17,570- $30,534). Moreover versus targeted therapies, immunotherapy recipients were more likely to suffer respiratory (76%), haematologic (71%) and gastrointestinal (79%) complications all of which costed more to manage ($21,041-$31,179) and led to greater 30 day treatment costs.^21^ This therefore represents a very real challenge for the NHS in advocating potentially life changing treatments and ensuring equal access while limiting their potential complications. In addition, despite nearly a decade of use many healthcare professionals are inexperienced in dealing with immunotherapeutics and their potential toxicities and as such often requires tertiary hospital specialist input and admission. This may not only affect service provision but could antagonise existing area specific health inequalities. This emphasises the need therefore to identify methods to risk stratify patients who will likely be older, suffered progressive disease despite past treatments with attendant comorbidities, organ dysfunction and reduced functionality which themselves may be complicated and exacerbated by immunotherapy.

This therefore is an important consideration for the NHS, a publicly funded body which has competing interests in managing the increasing demands from an ageing population and nation as a whole, in whom cardiovascular and metabolic disease is becoming more prevalent and for whom sustainability is key. This challenge however is not only restricted to the UK. In America, the cost of cancer care will reach between $173-206 billion this year and as a result of the emerging role of ‘maintenance’ cancer treatment, cancer is quickly becoming a chronic disease with costs set to rise.^2^ To address this one must first consider the challenges of economic modelling in modern oncology, given that for many therapies there is often limited trial data, diverse drug mechanisms of action and often limited applicability in only a subset of patients suffering a given cancer diagnosis, thereby hindering comparison. In addition, longterm data is often absent limiting one to accurately evaluate the total costs of treatment with quantitative quality of life data often not present which is an important consideration.^1^

Currently there are two main economic models which can be utilised to assess cost effectiveness. These are the Markov and Partitioned Survival (PS) Models. While the former considers patients to be in flux between different health states (i.e. progression free or progression of disease etc) and is based on assumptions of how likely such transitions are to take place; PS models rely on Kaplen-Meier survival curves and therefore are extrapolations of real life data.^22^ At present several outcome measures can be calculated from such models including quality adjusted life years (QALYs) which considers both quality and quantity of life (i.e. a QALY of 1 signifies 1 year of perfect health) and those costs associated with gaining an additional QALY the incremental cost effectiveness ratio (ICER) which is compared with a predetermined willingness to pay threshold. Comparison between these values can help demonstrate cost effectiveness. NICE traditionally have a QALY threshold of £20–30,000 which is substantially less than the USA threshold of $100,000. 23 During Ipilimumab’s appraisal NICE estimated the QALY to be ∼£240,000 with an ICER of £54,000–£70,000 while within NSCLC the ICER of Nivolumab and Pembrolizumab was $1,17,000 and $98,000 in primary and recurrent disease translating into a 3.5 month PFS.^13^ Importantly however when analysing the cost-effectiveness of Nivolumab according to tumour PD-L1 expression levels a trend to cost-effective satisfying the USA QALY threshold was seen, $1,12,311 (≥1%), $72,897 (≥5%), $78,921(≥10%) with similar results found with Pembrolizumab monotherapy. This therefore represents one method by which economic viability with immunotherapy use can be pursued.^20^ Regardless it is imperative that risk-sharing schemes are sought to both help mitigate these substantial costs and allow for their sustainable use. Given that even with the historical $50,000/QALY cited in the literature then only three of the twenty economic studies used in a 2018 systematic analyses of immunotherapies would demonstrate cost effectiveness.^23,24^ Importantly however with identification of suitable biomarkers and therefore improved patient selection, it can be deduced that QALY and ICER measures will decrease markedly while providing maximal clinical benefit.

In conclusion while immunotherapy offers to otherwise terminal patients a chance of robust durable responses it is associated with both high treatment costs and frequency of expensive adverse side effects which may limit its use. In addition, there is at present no method to differentiate between likely responders who represent the minority and non-responders which impacts their cost effectiveness. Therefore, future aims must be to identify suitable biomarkers and methods to enhance response rates while analysing cohort data to evaluate the long term clinical and financial outcomes of immunotherapy.

## References

[1] Kaufman HL Atkins MB Subedi P, et al The promise of immuno-oncology: implications for defining the value of cancer treatment. J Immunother Cancer 2019; 7: 129. 31101066 10.1186/s40425-019-0594-0 PMC6525438

[2] Kudrin A. Reimbursement challenges with cancer immunotherapeutics. Hum Vaccin Immunother 2012; 8: 1326–1334. 22894969 10.4161/hv.20550 PMC3579918

[3] Wilking N Bucsics A Kandolf Sekulovic L, et al Achieving equal and timely access to innovative anticancer drugs in the European union (EU): summary of a multidisciplinary CECOG-driven roundtable discussion with a focus on Eastern and South Eastern EU countries. ESMO Open 2019; 4: e000550. 31798977 10.1136/esmoopen-2019-000550 PMC6863652

[4] Tartari F Santoni M Burattini L, et al Economic sustainability of anti-PD-1 agents nivolumab and pembrolizumab in cancer patients: recent insights and future challenges. Cancer Treat Rev 2016; 48: 20–24. 27310708 10.1016/j.ctrv.2016.06.002

[5] Zhao Z, et al Delivery strategies of cancer immunotherapy: recent advances and future perspectives. J Haematol Oncol 2019; 12: 1–14 www.ncbi.nlm.nih.gov/pmc/articles/PMC6883629/ (accessed 2 June 2020). 10.1186/s13045-019-0817-3 PMC6883629 31779642

[6] Dobosz P Dzieciątkowski T. The intriguing history of cancer immunotherapy. Front Immunol 2019; 10: 2965. 31921205 10.3389/fimmu.2019.02965 PMC6928196

[7] June CH Warshauer JT Bluestone JA. Is autoimmunity the Achilles’ heel of cancer immunotherapy? Nat Med 2017; 23: 540–547. 28475571 10.1038/nm.4321

[8] Bugeon L Dallman MJ. Costimulation of T cells. Am J Respir Crit Care Med 2000; 162: S164–S168. 11029388 10.1164/ajrccm.162.supplement_3.15tac5

[9] Ugurel S Paschen A Becker JC. Dacarbazine in melanoma: from a chemotherapeutic drug to an immunomodulating agent. J Invest Dermatol 2013; 133: 289–292. 23318786 10.1038/jid.2012.341

[10] Hodi FS O'Day SJ McDermott DF, et al Improved survival with ipilimumab in patients with metastatic melanoma. N Engl J Med 2010; 363: 711–723. 20525992 10.1056/NEJMoa1003466 PMC3549297

[11] Hanauzu Z, et al The european medicines agency review of ipilimumab (yervoy) for the treatment of advanced (unresectable or metastatic) melanoma in adults who have received prior therapy: summary of the scientific assessment of the committee for medicinal products for human use. Eur J Cancer 2012; 48: 237–242. 22030452 10.1016/j.ejca.2011.09.018

[12] Bruce F. “Step change” Yervoy from BMS not cost effective for NICE: may need biomarker test, www.scripintelligence.com/home/Step-change-Yervoy-from-BMS-not-cost-effective-for-NICE-may-need-biomarker-test-322459 (2011, accessed 23 September 2020).

[13] Wolchok JD Chiarion-Sileni V Gonzalez R, et al Overall survival with combined nivolumab and ipilimumab in advanced melanoma. N Engl J Med 2017; 377: 1345–1356. 28889792 10.1056/NEJMoa1709684 PMC5706778

[14] Ventola CL. Cancer immunotherapy, part 3: challenges and future trends. PT 2017; 42: 514–521. PMC5521300 28781505

[15] Hryniewicki AT Wang C Shatsky RA, et al Management of immune checkpoint inhibitor toxicities: a review and clinical guideline for emergency physicians. J Emerg Med 2018; 55: 489–502. 30120013 10.1016/j.jemermed.2018.07.005

[16] Som A Mandaliya R Alsaadi D, et al Immune checkpoint inhibitor-induced colitis: a comprehensive review. World J Clin Cases 2019; 7: 405–418. 30842952 10.12998/wjcc.v7.i4.405 PMC6397821

[17] Kennedy LB Salama AKS. A review of cancer immunotherapy toxicity. CA Cancer J Clin 2020; 70: 86–104. 31944278 10.3322/caac.21596

[18] Lee EY Kulkarni RP. Circulating biomarkers predictive of tumour response to cancer immunotherapy. Expert Rev Mol Diagn 2019; 19: 895–904. 31469965 10.1080/14737159.2019.1659728 PMC6773262

[19] Kaufman HL Atkins MB Dicker AP, et al The value of cancer immunotherapy summit at the 2016 society for immunotherapy of cancer 31st anniversary annual meeting. J Immunotherapy Cancer 2017; 5: 1–10.

[20] Pereira da Veiga CR Pereira da Veiga C Drummon-Lage AP. Concern over cost of and access to cancer treatments: a Meta-narrative review of nivolumab and pembrolizumab studies. Crit Rev Oncol Hematol 2018; 129: 133–145. 30097232 10.1016/j.critrevonc.2018.07.002

[21] Ghate SR Li Z Tang J, et al Economic burden of adverse events associated with immunotherapy and targeted therapy for metastatic melanoma in the elderly. Am Health Drug Benefits 2018; 11: 334–343. 30647822 PMC6306100

[22] Verma V Sprave T Haque W, et al A systematic review of the cost and cost-effectiveness studies of immune checkpoint inhibitors. J Immunother Cancer 2018; 6: 128– 115. 30470252 10.1186/s40425-018-0442-7 PMC6251215

[23] Yu PP Eton O Garrison LP. Challenges in assessing the clinical utility and economic value of immune checkpoint inhibitor therapies of cancer. J Immunother Cancer 2019; 7: 235– 236. 31481112 10.1186/s40425-019-0707-9 PMC6724271

[24] Pai S, et al Defining current gaps in quality measures for cancer immunotherapy: consensus report from the society for immunotherapy of cancer (SITC) 2019 quality summit. J ImmunoTherapy of Cancer 2020; 8: 1–9. 10.1136/jitc-2019-000112 PMC7057483 31949040

